# DLEU1 contributes to ovarian carcinoma tumourigenesis and development by interacting with miR‐490‐3p and altering CDK1 expression

**DOI:** 10.1111/jcmm.13217

**Published:** 2017-06-09

**Authors:** Li‐Li Wang, Kai‐Xuan Sun, Dan‐Dan Wu, Yin‐Ling Xiu, Xi Chen, Shuo Chen, Zhi‐Hong Zong, Xiu‐Bo Sang, Yao Liu, Yang Zhao

**Affiliations:** ^1^ Department of Gynecology The First Affiliated Hospital of China Medical University Shenyang China; ^2^ Department of Biochemistry and Molecular Biology College of Basic Medicine China Medical University Shenyang China

**Keywords:** epithelial ovarian carcinoma, DLEU1, miR‐490‐3p, CDK1, tumourigenesis, development

## Abstract

Recently, a large number of studies have focused on the important role of long non‐coding RNAs (lncRNAs) in metabolism and development and have found that abnormal lncRNA expression is associated with the pathogenesis and development of many diseases. The lncRNA DLEU1 is involved in many solid tumours and haematological malignancies. However, its role in epithelial ovarian carcinoma (EOC) and the associated molecular mechanisms has not been reported. In this study, quantitative reverse transcription–PCR (qRT–PCR) demonstrated higher lncRNA *DLEU1* expression in EOC tissues than in normal tissues. Plasmid transfection of DLEU1 to up‐regulate its expression in the ovarian cancer cell lines A2780 and OVCAR3 increased cell proliferation, migration, and invasion, while inhibited apoptosis. Nude mouse xenograft assay demonstrated that DLEU1 overexpression promoted tumour growth *in vivo*. QRT–PCR showed decreased miR‐490‐3p expression, while Western blotting demonstrated increased its target genes *CDK1*, cyclinD*1* and *SMARCD1*, as well as matrix metalloproteinase‐2 (MMP2), Bcl‐xL and P70S6K protein expression, respectively. Short interfering RNA silencing of DLEU1 produced opposite results, where qRT–PCR showed increased miR‐490‐3p expression. The dual‐luciferase reporter assay revealed a direct interaction between DLEU1 and miR‐490‐3p. MiR‐490‐3p plays a tumour suppressor role in epithelial ovarian cancer by targeting CDK1 regulation and influencing SMARCD1 and cyclin D1 (CCND1) expressions. Therefore, we suggest that through interaction with miR‐490‐3p, DLEU1 may influence the expression of CDK1, CCND1 and SMARCD1 protein, subsequently promoting the development and progression of EOC.

## Introduction

EOC is the most common reason for reproductive system cancer‐related death; most patients are diagnosed at the late stage, and the 5‐year survival rate is <30% 1, 2, 3. The American Cancer Statistics 2016 show that there were more than 22,280 newly diagnosed cases of ovarian carcinoma in that year, which were associated with 14,240 deaths 4. Therefore, exploring the molecular mechanisms of the pathogenesis and development of ovarian cancer, as well as its therapeutic targets, are very important.

lncRNAs are a group of RNAs >200 nucleotides in length. As they lack a significant open reading frame, they cannot encode proteins 5, 6. In recent years, studies have shown that lncRNAs have powerful gene regulatory function and are involved in various pathophysiological processes 7, 8, and play an important role in cancer development 9, 10. LncRNA DLEU1, located on chromosome 13q14.3 11, is frequently knocked down in chronic lymphocytic leukaemia (CLL), multiple myeloma (MM) and other hematopoietic malignancies 12. In addition, *DLEU1* deletion has been found in atypical spindle cell lipoma 13, and *DLEU1* is highly expressed in breast cancer 14. In this study, we explored the role of *DLEU1* in EOC, and the corresponding molecular mechanism.

## Materials and methods

### Tissue specimens

EOC (benign ovarian tumours, *n* = 11; borderline ovarian tumours, *n* = 8, ovarian cancer, *n* = 99) specimens and normal ovary tissue (*n* = 15) were collected from patients undergone surgical resection at the Department of Gynecology of the First Affiliated Hospital of China Medical University (Shenyang, China). The tumour specimens were confirmed by two pathologists independently. All patients had not received preoperative radiotherapy or chemotherapy. The study was approved by the China Medical University Ethics Committee (No.2014‐27), all specimens were handled and made anonymous according to ethical and legal standards.

### Cell culture and transfection

The human ovarian carcinoma cell lines OVCAR3 and A2780 were cultured in RPMI 1640 (HyClone, Logan, UT, USA) or in Dulbecco's modified Eagle's medium (HyClone) with penicillin/streptomycin (100 U/ml) and 10% foetal bovine serum (FBS) supplemented in 5% CO_2_ and 37°C. The siRNA (sense: 5′‐GCAGUCUGUUCUGAACAUAdTdT‐3′ and anti‐sense:5′‐UAUGUUCAGAACAGACUGCdTdT‐3′) and DLEU1 plasmid (the details were shown in the Data S1) transfections were carried out using Lipofectamine 2000 (Invitrogen, Carlsbad, USA) following the manufacturer's instructions.

### MTT assay

A total of 3000 cells per well were seeded in 96‐well plates, and 20 μl MTT (5 mg/ml) was added to the wells at 0, 24, 48 and 72 hrs after transient transfection, which were incubated at 37°C for another 2~4 hrs. The medium was then removed and replaced by 150 μl dimethyl sulfoxide to dissolve the precipitated formazan. The absorbance at 490 nm was detected using a microplate spectrophotometer (BioTek Instruments, Winooski, VT, USA).

### Apoptosis assay

The cells were collected and washed with cold phosphate‐buffered saline (PBS). Then, for the cells transfected with si‐RNA, 100 μl 1× buffer plus 5 μl propidium iodide (PI) and 5 μl fluorescein isothiocyanate (FITC)‐labelled annexin V (BD Biosciences, San Jose, CA, USA) per sample were used to stain in the dark. And for the cells transfected with the plasmid, 7AAD and PE‐labelled annexin V (BD Biosciences) were used. Then 400 μl 1× buffer was added to each sample, the total apoptotic rate was determined by flow cytometry.

### Wound healing assay

Cells were seeded at 10^6^ cells/well in a 6‐well plate and cultured to 80% confluence. The monolayers were scratched with a 200‐μl pipette tip, and then the cells were cultured in FBS‐free medium. The wounds were observed under light microscopy and photographed at 0, 24 and 48 hrs. The nude areas were measured using Image J software (National Institutes of Health, Bethesda, MD, USA). The wound healing rate was calculated as follows: (area of original wound—area of wound at different time‐points)/area of original wound × 100%.

### Invasion assay

We used matrigel‐coated Transwell cell culture chambers (BD Bioscience) for the invasion assay. Filters were coated with 30 μl basement membrane Matrigel (1:10). Cells (5 × 10^4^/l) re‐suspended in 200 μl serum‐free medium were layered in the upper compartment of the Transwell inserts. The bottom chambers contained 600 μl complete medium serving as the chemo attractant. After 48‐hrs incubation at 37°C, cells and matrigel on the upper surface of the filter were removed, and the invaded cells were fixed with formaldehyde, stained with crystal violet and counted under an Olympus fluorescence microscope (Tokyo, Japan).

### Real‐time RT‐PCR

Total RNA was isolated from the EOC cell lines and tissues using TRIzol (Takara, Shiga, Japan) and was reverse‐transcribed to complementary DNA (cDNA) using avian myeloblastosis virus transcriptase and random primers (Takara) according to the manufacturer's protocol. The cDNA was amplified by real‐time qPCR using a SYBR Premix Ex Taq II kit (Takara). The expression level of each target gene was normalized to that of *18S* mRNA. Data were analysed according to the sample threshold cycle (Ct) value from three independent experiments.

### Western blotting

Total cell proteins were lysed in radioimmunoprecipitation assay buffer containing protease inhibitors. All protein samples were separated on 10% sodium dodecyl sulphate–polyacrylamide gels and electro‐transferred to Hybond membranes (Amersham, Munich, Germany) at the concentrations of 1 mg/ml. Fat‐free milk (5%) was used to block the membranes for 2 hrs at room temperature. Subsequently, primary antibodies against CDK1, SMARCD1, CCND1, P70S6K, MMP2 and Bcl‐xL (Proteintech, Chicago, IL, USA) were incubated with the blots overnight at 4°C. Then the membranes were incubated with the secondary antibody for 2 hrs at room temperature after removing the primary antibodies and washed three times with TBST. An enhanced chemiluminescence system was used to visualize the proteins following the manufacturer's protocol (Santa Cruz Biotechnology, Santa Cruz, CA, USA).

### *In vivo* nude mouse xenograft assay

All animal experiments were undertaken according to the National Institutes of Health Guide for the Care and Use of Laboratory Animals, and with the approval of the China Medical University Animal Care and Use Committee. Female BALB/c mice (4‐week‐old) obtained from Vital River Laboratories (Beijing, China) were routinely housed in temperature‐ and light‐controlled (12‐hrs dark/light) rooms. The animals had free access to food and water. A2780 cells transfected with mutant or wild‐type DLEU1 (1 × 10^7^ cells per type) were re‐suspended in 200 μl FBS‐free culture medium and injected subcutaneously into the right flanks of the mice. The tumour volume was directly measured following inoculation, and tumour weights were calculated using the following formula: $(\text{length}\times {\text{width}}^{2})/2.$

### Immunohistochemistry

Paraffin‐embedded tissue sections for detecting the expression of CDK1 were deparaffinized in xylene, and then rehydrated in a graded series of ethanol solutions (95%, 85%, 75%), and then 3% H_2_O_2_ incubated the samples which was used to quench the endogenous peroxidase activity. Next, the sections were heated in target retrieval solution (Dako) for antigen retrieval. Non‐specific binding was blocked by 10% goat serum for 2 hrs at room temperature. The samples were then probed overnight at 4°C with anti‐CDK1 primary antibody (1:100). An appropriate secondary antibody (1:200) was added, and the slides were incubated for 20 min. at 37°C and then were visualized with 3, 39‐diaminobenzidine tetrahydrochloride staining.

### Statistical analyses

Statistical analyses were performed using SPSS 17.0 (SPSS, Chicago, IL, USA). The Spearman correlation test was used to analyse the rank data; the Student's *t*‐test was used to differentiate the means of two groups. All data shown are the mean ± standard deviation of at least three separate experiments. *P *<* *0.05 was considered statistically significant.

## Results

### DLEU1 expression is associated with EOC tumourigenesis and progression

**Figure 1:**
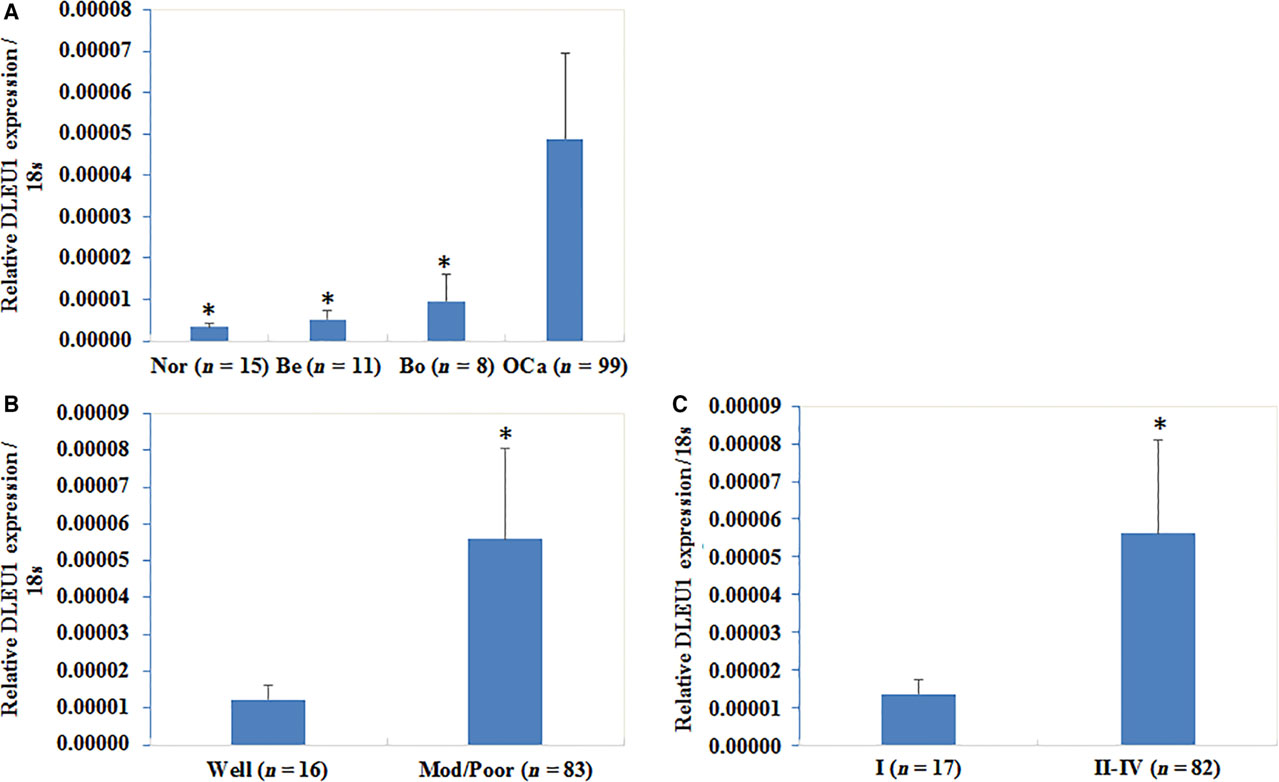
Correlation of lncRNA DLEU1 expression with pathogenesis and aggressiveness of epithelial ovarian carcinoma. Our RT‐PCR results demonstrated that DLEU1 was overexpression in ovarian cancer tissues than normal ovarian tissues, benign ovarian tumours and borderline ovarian tumours (**A**), nor = normal ovarian tissues, be = benign ovarian tumours, bo = borderline ovarian tumours, OCa = ovarian cancer tissues; *, V.S. OCa, *P* < 0.05). DLEU1 expression was positively associated with differentiation (well *versus*. poor and moderate, (**B**), *P* < 0.05) as well as International Federation of Gynecology and Obstetrics (FIGO) staging (stage I *versus*. stage II/III/IV, (**C**), *P* < 0.05).

qRT–PCR showed significantly higher lncRNA DLEU1 expression in EOC tissues than in normal ovarian tissues, benign ovarian tumours and borderline ovarian tumours (Fig. 1A, *P* < 0.05), and lncRNA DLEU1 expression was positively associated with differentiation (well *versus*. poor and moderate, Fig. 1B, *P* < 0.05), and International Federation of Gynecology and Obstetrics (FIGO) staging (stage I *versus*. stage II/III/IV, Fig. 1C, *P* < 0.05). The details were shown in the Table S1 and S2.

### DLEU1 expression affects EOC cell proliferation

**Figure 2:**
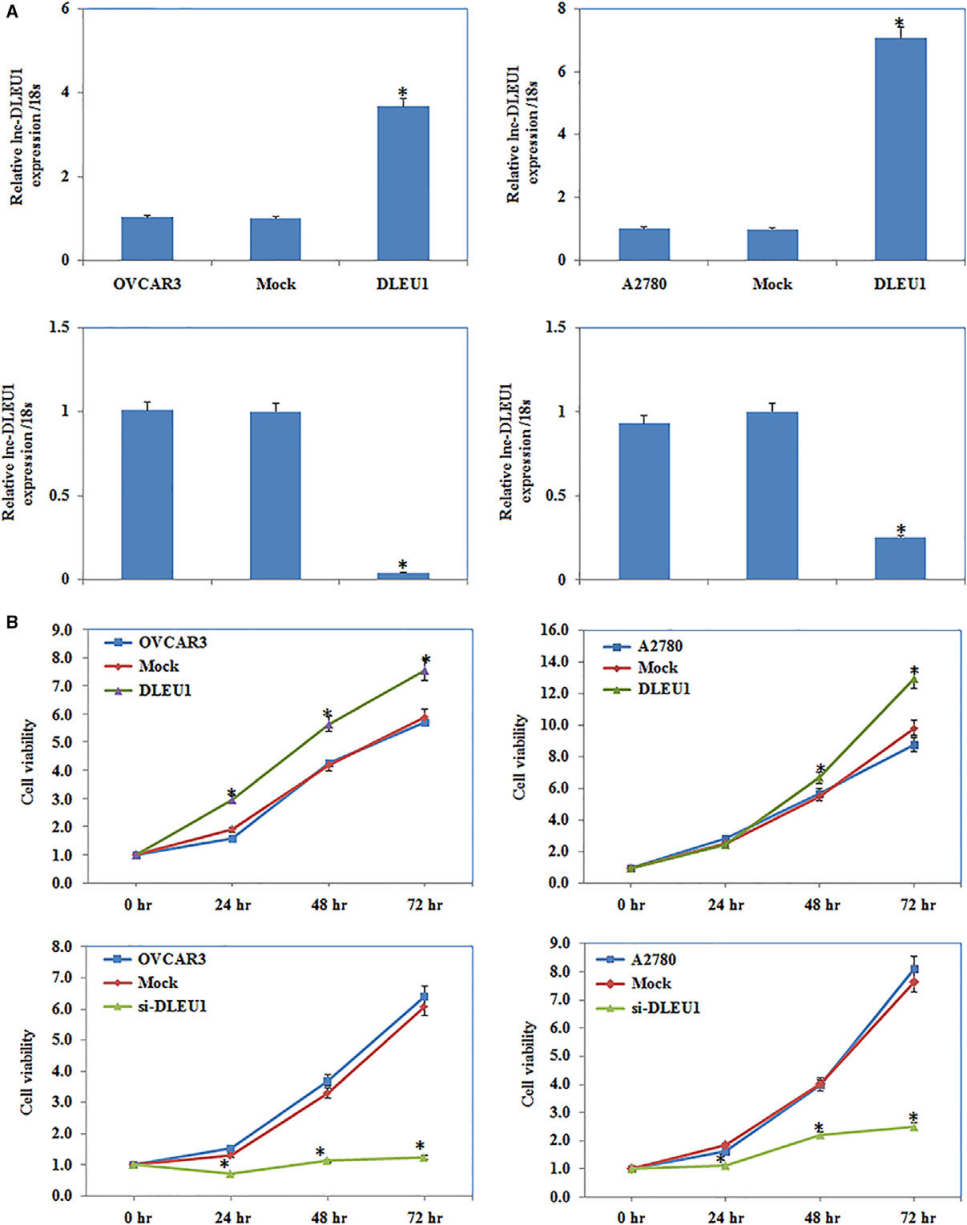
The effect of DLEU1 expression on ovarian carcinoma cell proliferation. QRT–PCR analysis showed that DLEU1 si‐RNA reduced lnc‐DLEU1 expression and plasmid transfection induced lnc‐DLEU1 expression (**A**). DLEU1 overexpression increased cell viability compared with control and mock‐transfected cells, and silencing expression of DLEU1 with si‐RNA‐reduced cell viability by MTT (**B**). Results are representative of three separate experiments; data are expressed as the mean ± standard deviation, *P < 0.05.

The qRT–PCR detected increased or decreased *DLEU1* expression after plasmid or si‐RNA transfection (Fig. 2A). Cell proliferation ability was significantly increased after DLEU1 overexpression. Silencing DLEU1 with small interfering RNA (siRNA) reduced cell viability significantly (*P *<* *0.05; Fig. 2B), as determined using the 3‐(4,5‐dimethylthiazol‐2‐yl)‐2,5‐diphenyl tetrazolium (MTT) proliferation assay.

### DLEU1 expression affects EOC cell apoptosis

**Figure 3:**
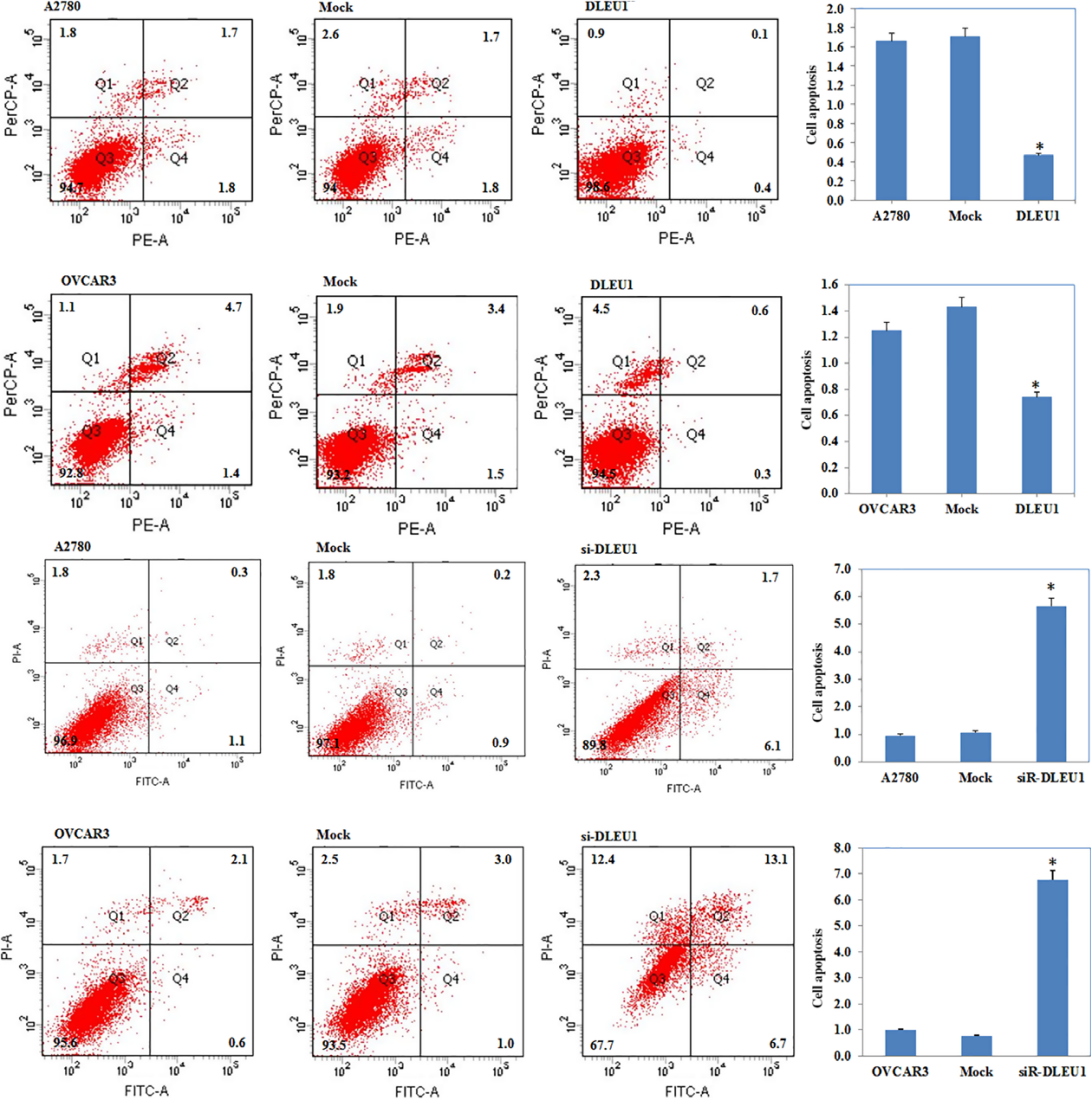
The effect of DLEU1 expression on ovarian carcinoma cell apoptosis DLEU1 overexpression reduced cell apoptosis compared with the control and mock‐transfected cells, and si‐DLEU1‐induced early‐stage apoptosis of ovarian carcinoma. Results are representative of three separate experiments; data are expressed as the mean ± standard deviation, *P < 0.05.

Flow cytometry demonstrated that *DLEU1* up‐regulation induced lower levels of apoptosis compared to the control and mock‐transfected cells. Silencing *DLEU1* with siRNA obviously increased the percentage of cells in early apoptosis 48 hrs after transfection (*P *<* *0.05; Fig. 3).

### Changes in EOC cell migration and invasive ability

**Figure 4:**
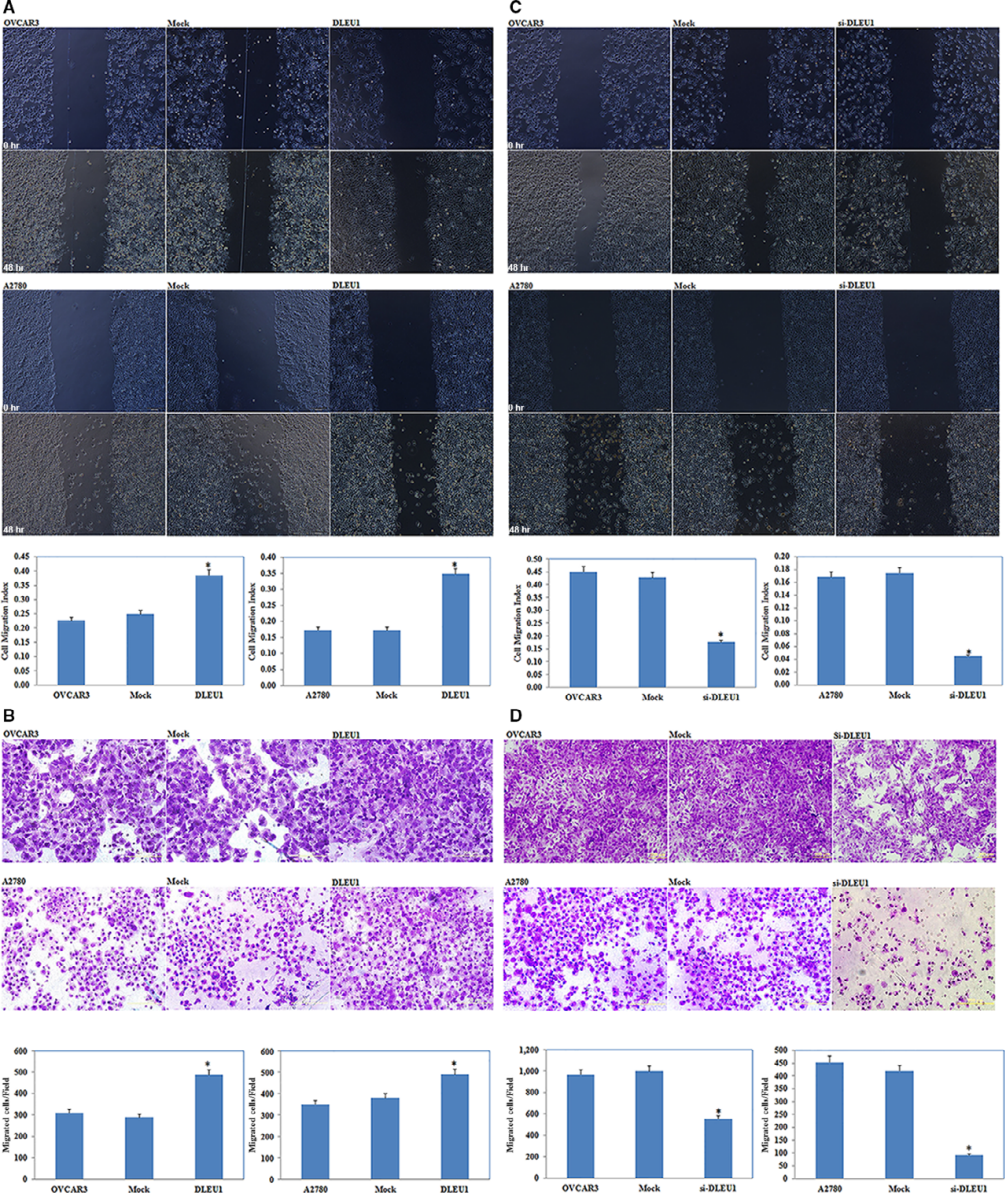
The effect of DLEU1 expression on ovarian carcinoma cell migration and invasion Would healing assay showed that DLEU1 overexpression induced cell migration ability compared with the control and mock‐transfected cells (**A**). And the cells transfected with DLEU1 demonstrated higher invasion ability compared with control and mock‐transfected cells (**B**). DLEU1 downregulation reduces ovarian carcinoma cell migration and invasion.(**C** and **D**) Results are representative of three separate experiments; data are expressed as the mean ± standard deviation, *P < 0.05.

The wound healing assay showed that DLEU1 overexpression increased cell migration (*P *<* *0.05; Fig. 4A); Transwell assay evaluation of cell invasive ability showed significant induction (Fig. 4B, *P* < 0.05) compared to the control and mock‐transfected groups. DLEU1 down‐regulation reduced EOC cell migration and invasive ability (*P *<* *0.05; Fig. 4C and D).

### Up‐regulated DLEU1 promotes EOC tumourigenesis *in vivo*

**Figure 5:**
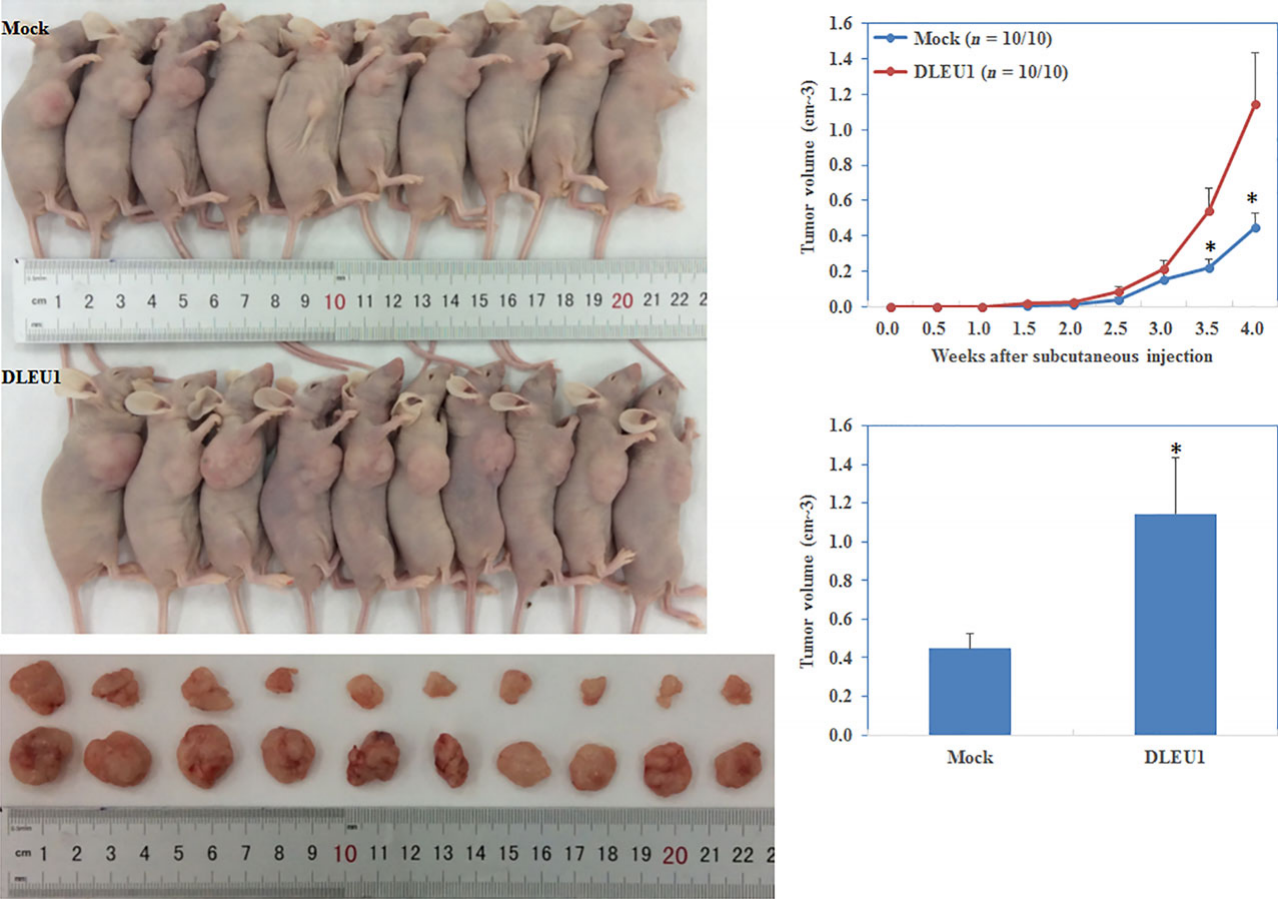
DLEU1 overexpression induces the tumourigenicity of ovarian carcinoma cells *in vivo* DLEU1 transfection induced tumourigenicity after inoculation compared with the control group and exhibited a bigger tumour volume given the same duration. *P <* *0.05.

The nude mouse xenograft assay demonstrated that the tumours formed after implantation with DLEU1‐transfected cells were of greater volume than that of the control group during the same observation period (*P *<* *0.05; Fig. 5).

### DLEU1 interacts with miR‐490‐3p

**Figure 6:**
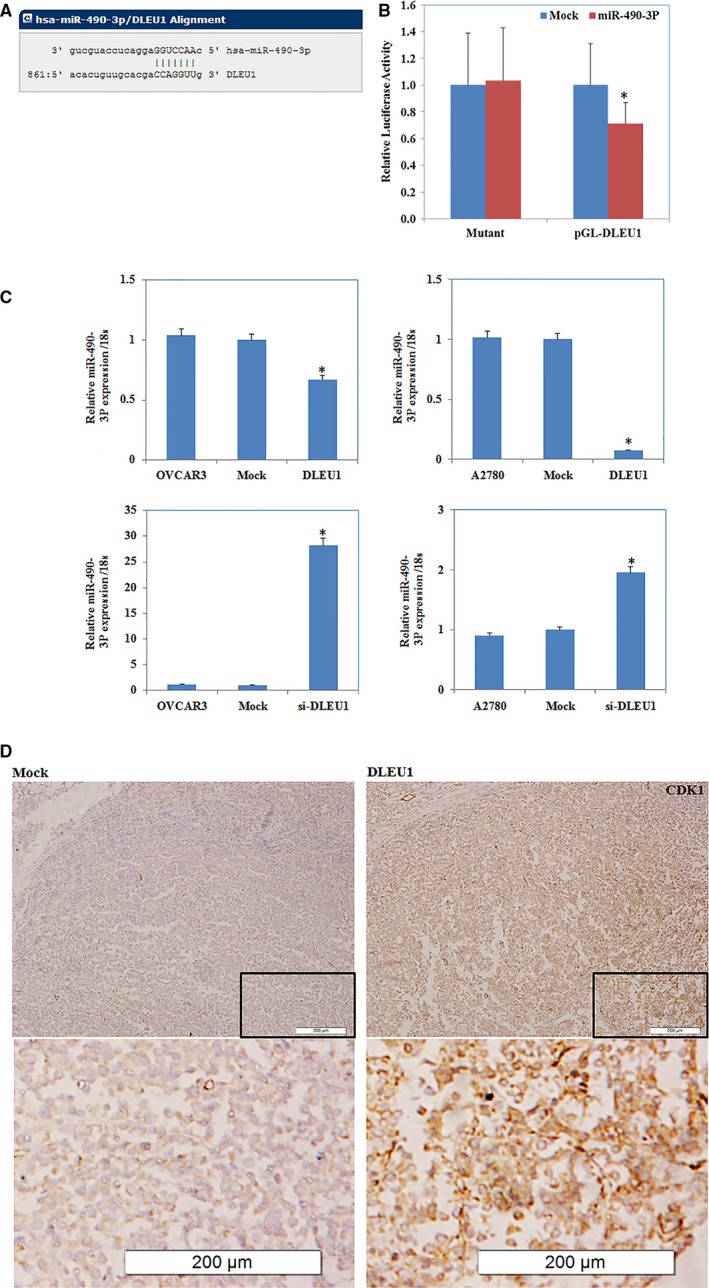
DLEU1 interacted with miR‐490‐3p in ovarian carcinoma A target microRNA (miRNA) prediction website and dual‐luciferase reporter assay showed the biding site between DLEU1 and miR‐490‐3p(**A** and **B**). Down‐regulated expression of DLEU1 increased the miR‐490‐3p mRNA lever and overexpression of DLEU1 reduced miR‐490‐3p mRNA expression(**C**). The up‐regulated expression of DLEU1 increased the expression of CDK1, miR‐490‐3p target protein levels (**D**). *P < 0.05.

**Figure 7:**
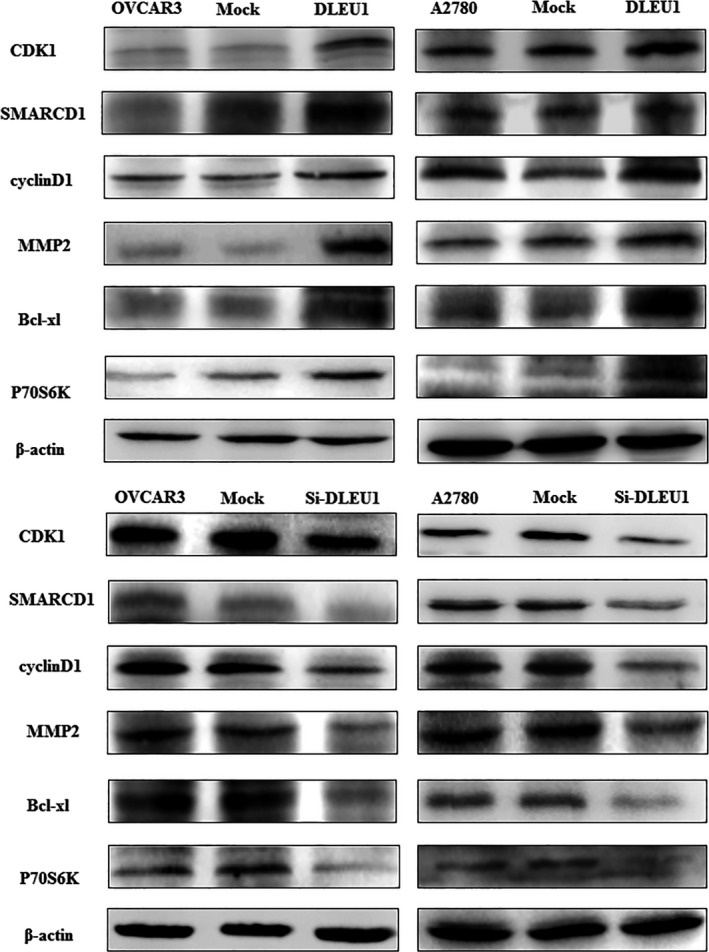
DLEU1 expression regulates CDK1, SMARCD1, cyclinD1, MMP2, Bcl‐xl and P70S6K proteins expression. Western blot results showed protein expression of CDK1, SMARCD1, cyclinD1, MMP2, Bcl‐xl and P70S6K were increased with the overexpression of DLEU1 but the protein expression level above decreased by si‐DLEU1.

A target microRNA (miRNA) prediction website (http://microRNA.org) and the luciferase reporter assay showed that there was a binding site between DLEU1 and miR‐490‐3p (*P *<* *0.05; Fig. 6A and B). QRT–PCR showed that *DLEU1* transfection reduced miR‐490‐3p expression (*P *<* *0.05; Fig. 6C). Immunohistochemical analysis of tumour tissues from the nude mice demonstrated significant induction of CDK1 expression in the DLEU1 transfection group compared to the control group (*P *<* *0.05; Fig. 6D). The up‐regulated expression of DLEU1 in the EOC cells increased the expression of *CDK1*, cyclin*D1* and *SMARCD1*, the target genes of miR‐490‐3p, at protein level. Treatment with DLEU1 siRNA (si‐DLEU1) yielded opposite results (*P *<* *0.05; Fig. 7). Taken together, these results suggest that DLEU1 interacts with miR‐490‐3p.

### Expression of P70S6K, Bcl‐xL and MMP2 proteins is altered according to DLEU1 up‐regulation or down‐regulation

Western blotting showed increased P70S6K, MMP2 and Bcl‐xL proteins expression after DLEU1 up‐regulation in EOC cells. Conversely, siRNA knock down of DLEU1 reduced MMP2, Bcl‐xL and P70S6K proteins expression (*P *<* *0.05; Fig. 7).

## Discussion

Currently, cancer is one of the most important threats to human health and survival. Exploration of the pathogenesis and development mechanisms of cancer is very important, and the role played by lncRNAs therein has attracted widespread attention. Recent studies have found that lncRNAs are involved in a wide range of biological processes, including proliferation, the cell cycle, apoptosis, differentiation and maintenance of pluripotency 6. In solid tumours and haematopoietic malignancies such as CLL, there is recurrent deletion of *DLEU1*; Dowd *et al*. detected 13q14 deletion in six patients with MM (54.5%), while one patient (9.1%) showed monosomy; all relapsed MM cases (27.3%) had 13q14 deletion. In mycosis fungoides tumour samples, deletion of the tumour suppressor gene *DLEU1* is one of the reasons for tumourigenesis 15. Wu *et al*. found that DLEU1 is highly expressed in the oestrogen receptor (ER)‐positive breast cancer cell line MCF‐7.

To explore the role of DLEU1 in ovarian cancer, we examined its expression in normal ovary and ovarian cancer tissue samples, and found that DLEU1 expression was significantly higher in the ovarian cancer tissues than in the normal tissues. Stable transfection of DLEU1 in two ovarian cancer cell lines (A2780 and OVCAR3) promoted proliferation, migration and invasion, while inhibiting apoptosis. Silencing DLEU1 expression with siRNA yielded opposite results. In addition, stable transfection of DLEU1 accelerated nude mouse xenograft growth. These results demonstrate that DLEU1 plays an oncogenic role in EOC pathogenesis and development, which is consistent with the findings of Wu *et al*. in breast cancer.

In recent years, many studies have found a complex signalling network between lncRNAs and miRNAs in a variety of physiological and pathological processes. LncRNAs may act as a competing endogenous RNA (ceRNA) or molecular sponge, regulating miRNA expression and biological function 16, 17. *Via* negative regulation of miR‐21, the lncRNA CASC2 plays a tumour‐suppressive role in glioma 18. In human muscle cells, linc‐MD1 serves as a ceRNA of miR‐133, where the ceRNA network plays an important role in muscle differentiation 19. DLEU1 is up‐regulated in MCF‐7 cells and might be coexpressed with miR‐19a to co‐regulate the expression of ER‐α (*ESR1*), which influences the occurrence and development of breast cancer. In the present study, bioinformatic predictions and dual‐luciferase reporter assays revealed a binding site between DLEU1 and miR‐490‐3p; qRT–PCR demonstrated increased miR‐490‐3p expression after DLEU1 had been silenced. Therefore, we believe that DLEU1 and miR‐490‐3p interact in ovarian cancer.

MiR‐490‐3p has been confirmed as a tumour suppressor in many cancers. Zhao *et al*. found that miR‐490‐3p inhibits cell proliferation in breast cancer 20, and miR‐490‐3p overexpression inhibits proliferation in the lung cancer A549 cell line 21. In addition, miR‐490‐3p up‐regulation suppresses the development and regeneration of murine embryonic stem cells 22. Previously, we showed that miR‐490‐3p targets CDK1 and down‐regulates CCND1 and SMARCD1 protein expression in ovarian cancer, plays the role of a tumour suppressor gene 23.

We found that up‐regulated DLEU1 expression increased CDK1, CCND1 and SMARCD1 protein expression and that si‐DLEU1 produced opposite results. We suggest that DLEU1 affects the expression of its target genes by regulating the expression of miR‐490‐3p. CDK1, a cell cycle regulator CDK family member, is the key molecule of the ATM‐chk2‐CDC25‐CCNB1/CDK1 pathway that controls the cell cycle 24, 25. Xi *et al*. showed that CDK1 expression is associated with ovarian cancer cell proliferation and can serve as an independent prognostic factor 26. CCND1 stimulates G1 progression in responding to growth factor stimulation 27; miR‐211 down‐regulation results in CCND1 and CDK6 overexpression, which increases the proliferative ability of EOC cells 28.SMARCD1, whose high expression predicts poorer prognosis in gastric cancer, functions as an oncogene by promoting gastric cancer cell proliferation, migration and invasion 29. In the present study, up‐regulating DLEU1 increased MMP2, Bcl‐xL and P70S6K protein expression significantly. Western blotting showed opposite results following siRNA silencing of DLEU1 expression. Bcl‐xL up‐regulation and down‐regulation inhibits and promotes apoptosis, respectively, while MMP2 plays a key role in cell invasion and P70S6K together with its downstream effector S6, were initially identified as a key player, in the regulation of cellular growth and survival.

In summary, we believe that DLEU1 promotes the pathogenesis and development of epithelial ovarian cancer through its interaction with miR‐490‐3p, thereby altering the expression of the miR‐490‐3p target genes, that is, *CDK1*,* CCND1* and *SMARCD1*, as well as the expression of MMP2, Bcl‐xLand P70S6K. Our study clarifies the occurrence and possible molecular mechanisms of EOC and provides new perspectives for early diagnosis and treatment. The interactions and molecular mechanisms between lncRNAs and miRNAs warrant further exploration.

## Conflicts of interest

The authors have no conflicts of interest to declare.

## Supporting information


**Table S1** DLEU1 expression in normal ovary and ovarian carcinoma tissues
**Table S2** Correlation of DLEU1 expression with different clinicopathological features of ovarian carcinoma.
**Data S1** DLEU1 Vector construction.
