# Supplementary material for: DLEU1 contributes to ovarian carcinoma tumourigenesis and development by interacting with miR‐490‐3p and altering CDK1 expression
Source: J Cell Mol Med. 2017 Jun 9;21(11):3055–65. doi: 10.1111/jcmm.13217 (PMC5661118; doi:10.1111/jcmm.13217)
Supplement: Supplementary file 1 — Table S1 DLEU1 expression in normal ovary and ovarian carcinoma tissues Table S2 Correlation of DLEU1 expression with different clinicopathological features of ovarian carcinoma. Data S1 DLEU1 Vector construction. [file JCMM-21-3055-s001.doc]

**Supplementary Table 1:** DLEU1 expression in normal ovary and ovarian carcinoma tissues

| **Groups** | **N** | **DLEU1 expression / 18s** | ***P* value** |
| --- | --- | --- | --- |
|
| Normal ovary | 15 | 3.31E-06 ± 4.30E-06 | ***0.015*** |
| Benign ovarian tumors | 11 | 5.16E-06 ± 6.68E-06 | ***0.019*** |
| Borderline ovarian tumors | 8 | 9.54E-06 ± 1.84E-05 | ***0.037*** |
| Ovarian carcinoma | 99 | 4.88E-05 ± 2.06E-04 |  |
|  |  |  |  |

Bold and Italics means P < 0.05.

**Supplementary Table 2:** Correlation of DLEU1 expression with different clinicopathological features of ovarian carcinoma

| **Clinicopathological features** | **N** | **DLEU1 expression / 18s** | ***P* value** |
| --- | --- | --- | --- |
|
| **The pathology types** |  |  | 0.486 |
| Serous carcinoma | 76 | 4.85E-05 ± 2.26E-04 |  |
| The other pathology types | 23 | 4.98E-05 ± 1.23E-04 |  |
| **Age** |  |  | 0.146 |
| ≤ 52 | 50 | 2.68E-05 ± 7.61E-05 |  |
| > 52 | 49 | 7.13E-05 ± 2.82E-04 |  |
| **FIGO stages** |  |  | ***0.048*** |
| I | 17 | 1.37E-05 ± 1.58E-05 |  |
| II-IV | 82 | 5.61E-05 ± 2.26E-04 |  |
| **Pathology classification** |  |  | ***0.042*** |
| Well | 16 | 1.24E-05 ± 1.45E-05 |  |
| Mod + Poor | 83 | 5.58E-05 ± 2.24E-04 |  |
| Bold and Italics means P < 0.05. | | | |

**Supplementary Table 3: DLEU1 Vector construction**

TGGCTTATCGAAATTAATACGACTCACTATAGGGAGACCCAAGCTGGCTAGCGTTTAAACGGGCCCTCTAGACTCGAGAGGCACATGCGCAGAATCATCGTGGTGCACGGCTCTCCCTTTGCTTCTTCGGTTGCAGTCCTCTTGCTTCTTGCGCGTGCGTGTAGCGCTTTTGCAAAGCCGCGGAGGTGAAGTGAACTTAGAGGTTGTGGGGCCGAGGGGTCGTCTTATAGCTACCAGCCCACAGGCATTTAGTCTACGTTGGAGGTAAACAAATACGGGTCCTGCTTAGGAGAAAAGAAAAACGTCTTACAGCCAGTGTCTAAACTCCAAACAACGGAATGTATCAATGAGACCTTGTATATGGATACACGTGCATTTAAAACCGCCCTGCCGGCTTGTAGAGCTTTTGCCGTTCTCCAGCGCTTTACAGGGGTTATCGCACTTAAGCCTCGGAACAACTTTACCAGATGAGGACACCTGAGGTTCAGATTAAGAAATCTGCCCCAAAGTCTTAGAACTGGTATTCTCCACTGGTTGAATGCAAATGGAAGCCAGAGGAAAAGGGAACCCTTGACACAGTTTTCATATAGATTGGCCTCCAGGGCAGACAGCAGTTGCTCTGGCTATATCAAATAAAAGTGTCAAGAGTGAGCATCCTTGCCTTGTGCTGAATCACAAAGGAATACCTTTCAGTTTTTCTCCATTGATTATGATAGCAGTGGGCTTTTCACAGTGGGCTTTACTGTGTTGAGGTACCCTAATGACAAGTTCACCCCAGTGTTTTCTGAGGAGTCTACCTGGAAATGAGAACCATCTGTCCTTCCTATAGTAGTTCTTCTCAGAATGCCGACTCTATGCTAATGGAGGAGGTCTGATGGCCGGGAGGACAGGAAGAAGGAACCCACAATGAAGCAAAAGTTGCGTTTAACTCTGTCCTCACCATTCCTCTCCTGGACCTGTCCTAAACTGGAAGCTGGGGGAGGATGGAGTGCTGGAATGCTGCAGAAGTCTCATGGGAGGGCGCCTGGTGTTCACCACATGTGTACACTGTTGCACGACCAGATGGAAATTTGAATGCTGGGGCTCAGTGGAACACCTGAAGGAAGCTGGTGAGGCAGATGTCCCGCTATGCCTTCCAAGTGTCATGCAACCAGTTCATGCCTGTCAGCATCTCTGCCGGTGGACTCTGTGAGTCAGCAAATCTACAGAGCAGAGAGCCACAAGTGGGGACAGATGTGGTCAAATGCCATTTAAATCAGTGATTCTCAACCCAGGAGTTGTCATGGCACTGGCCTTGAGTGAGCTGGACCAAGGGAAGTGGTAGAATTCCCCATGAAGGACCAAGTGAGCAGATAGAAGTTTCTCCATTTTAACTTTTTCTGAGTCAATTTAAAAAACACCTAGTGCCTTTTTCTGTACATAGTGCCTGTGGGTTTGAATTTAAGCCTACCCAAATGTGCTTTTATCCCACCTATTTTGAACCTTGCATGTCTCCGGGATAAGAATTAACATAAGCTATAAATATTGGTACCAAGCTTAAGTTTAAACCGCTGATCAGCCTCGACTGTGCCTTCTAGTTGCCAGCCATCTGTTGTTTGCCCCTCCCCC
